# Supplementary material for: SPECTRE: a suite of phylogenetic tools for reticulate evolution
Source: Bioinformatics. 2017 Nov 24;34(6):1056–7. doi: 10.1093/bioinformatics/btx740 (PMC5860355; doi:10.1093/bioinformatics/btx740)
Supplement: Supplementary Data [file btx740_supplementary.pdf]

Spectre: Supplementary Material

Sarah Bastkowski, Daniel Mapleson, Andreas Spillner,
Taoyang Wu, Monika Balvočiūtė and Vincent Moulton

October 13, 2017

1 Tools

Documentation is provided in multiple forms, including useful command line help and error messages, tooltips for graphical front ends and an extensive technical manual, which is provided with the software and independently hosted online at: `http://spectre-suite-of-phylogenetic-tools-for-reticulate-evolution.readthedocs.io/en/latest/`.

The main graphical interface for the software is accessed via SPECTRE. From here you can visualise and modify trees and networks and launch most of the main tools within the toolkit.

1.1 NetMake

Netmake rapidly creates a circular split system producing an outer-planar network from:

1. Multiple sequence alignment
2. Distance matrix

It allows the user to select either NeighborNet (Bryant and Moulton, 2004) or variations of NeighborNet (Levy and Pachter, 2011) for constructing the circular ordering of taxa and can be used with large numbers of taxa.

1.2 FlatNJ

FlatNJ creates a flat planar split network where labels are not forced to the edges of the network (Balvočiūtė *et al.*, 2014), often producing richer and less distorted networks than Neighbornet, if supported by the data. FlatNJ however cannot be driven from a distance matrix alone, requiring quadruples (weighted collections of 4 taxa) instead. This comes at some additional computational cost, as does computation of edge weights, which is performed via solving an optimisation problem. FlatNJ can automatically generate quartets from the following input types:

1. Multiple sequence alignment
2. Geographical coordinates
3. Weighted split system

1.3 SuperQ

SuperQ computes a phylogenetic super network, in the form of a circular split system, from a collection of partial input trees (Grünewald *et al.*, 2013). SuperQ also uses collections of 4 taxa as an intermediary format, from which it uses QNet (Grünewald *et al.*, 2007) to create the initial super network. Weights for edges in the super network are calculated through solving an optimisation problem. SuperQ supports a variety of alternative input types which eventually get converted into collections of quartets:

1. A set of partial trees
2. A set of split systems
3. A set of distance matrices

1.4 NetME

NetME finds the minimum evolution tree within a circular split system (Bastkowski *et al.*, 2014) such as those generated by NeighborNet or SuperQ.

2 Library

The tools described above make use of core library functionality where possible, promoting a robust modular design and consistency between tools. The core library is available for download and integration into third party java projects via Maven: `https://mvnrepository.com/artifact/uk.ac.uea.cmp.spectre/core`.

The core library contains the following data structures:

- Distance matrices
- Split Systems
- Quartets
- Trees
- Networks

There are also implementations of the following algorithms:

- Distance Calculators: Uncorrected hamming; Jukes Cantor; Kimura2 (K80)
- Circular Ordering: NeighborNet; NeighborNet variants
- Quadruple System Convertors: MSA; Locations; Split Systems
- Quartet System Convertors: Distances; Split Systems
- Planar Split Network Drawing Algorithm (supports drawing of Compatible, Circular and Flat Split Systems).

In addition we provide a number of file parsers:

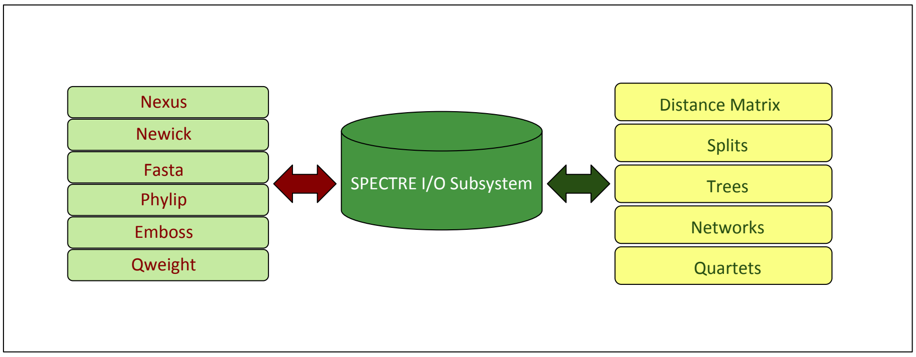

Figure 1: SPECTRE provides a number of file parsers that can handle commonly used formats and translates them into the implemented data structures as illustrated.

- Emboss distmat files
- FastA
- Newick
- Nexus (support for the following blocks: Taxa; Characters; Distances; Splits; Quartets; Quadruples; Locations; Assumptions; Trees; Networks; Spectre Viewer Config)
- Phylip
- QWeight (QNet format).

For each file format we capture the kinds of information that format can take, we then use this information within Spectre's IO subsystem to enable the user to load a particular data type from any supported file format. In this way the IO subsystem is written in an extendible way so as additional file format parsers can be added to the core library, the developer does not need to change application code in order to benefit from the new format. This concept is illustrated in section 2.

3 Mathematical Optimisation Module

Several tools in Spectre require solving linear and quadratic optimisation problems. Early versions used a commerical solver called Gurobi (`http://www.gurobi.com`), which is free to use under an academic license. However, to ensure that Spectre can be used freely, we produced another library enabling us to define both linear and quadratic programming problems via a common interface. These problems are then translated into solver specific definitions required by third party solvers. We currently support Gurobi, JOptimizer (`http://www.joptimizer.com/index.html`), Apache commons math (`http://commons.apache.org/proper/commons-math`) and GLPK (`http://www.gnu.org/software/glpk/`). This solution also allows researchers to compare solvers in terms of results and runtime performance.

The Spectre core library has no dependencies on the optimisation module or external optimisation systems in order to maximise portability of the library. Therefore each tool's module has a dependency on this optimisation module instead. The optimisation module is available from: `https://github.com/maplesond/metaopt`.

4 Addition of new tools

For the addition of new tools the recommended procedure would be to fork the GitHub repository and submit pull requests back to our repository. New tools should be added as a seperate module into SPECTRE with their own command line interface. If the tools come with a graphical user interface they should supply a Java Swing Frame, and we can then integrate this into the SPECTRE GUI. The specifics of where and how to make changes may vary from project to project and can be discussed via the pull request mechanism or through direct email with the developers.

References

Balvočiūtė, M., Spillner, A. and Moulton, V. (2014). FlatNJ: A novel network-based approach to visualize evolutionary and biogeographical relationships. *Systematic Biology*, **63**(3), 383–396.

Bastkowski, S., Spillner, A. and Moulton, V. (2014). Fishing for minimum evolution trees with NeighborNets. *Information Processing Letters*, **114**(1), 13–18.

Bryant, D. and Moulton, V. (2004). Neighbor-Net: An agglomerative method for the construction of phylogenetic networks. *Molecular Biology and Evolution*, **21(2)**, 255–265.

Grünewald, S. et al (2007). QNet: An agglomerative method for the construction of phylogenetic networks from weighted quartets. *Molecular Biology and Evolution*, **24**, 532–538.

Grünewald, S. et al (2013). SuperQ: Computing supernetworks from quartets. *IEEE/ACM Transactions on Computational Biology and Bioinformatics*, **10**(1), 151–160.

Levy, A. and Pachter, L. (2011). The Neighbor-Net algorithm. *Advances in Applied Mathemathics*, **47**(2), 240–258.
